# Resection of thoracic meningioma with hoth augmented reality system: technical note and operative video

**DOI:** 10.1007/s00701-025-06659-5

**Published:** 2025-09-12

**Authors:** Naser Ibrahim, Shuhei Shiino, Kenneth Nguyen, Stephen Shapiro, Andrew Janssen, Aaron Dumont

**Affiliations:** 1 Tulane Medical Center, Department of Neurosurgery, 131 S. Robertson St, Ste 1300, New Orleans, LA 70112 USA; 2 Department of Neurosurgery, Ochsner Health System, 131 S. Robertson St, Ste 1300, New Orleans, LA 70112 USA; 3 School of Medicine, Tulane University, New Orleans, LA USA

**Keywords:** Augmented reality, Neuronavigation, Spine surgery, Thoracic meningioma, Surgical video

## Abstract

**Background:**

Augmented Reality (AR) is an emerging technology in the field of neurosurgery. It allows surgeons to view three-dimensional (3D) patient anatomy overlaid onto the surgical field in real time. In this technical report, we describe the preoperative and intraoperative use of the Hoth AR system for resection of a thoracic meningioma.

**Methods:**

Prior to surgery, the 3D model of the patient’s tumor and surrounding anatomy is rendered from the patient’s preoperative imaging, and it is uploaded into the AR headset. Once the patient is positioned in the operating room, registration is performed by tracing reference points on the skin while wearing the headset. The surgeon wears the headset while operating, and they can toggle on–off and transparency of each component of the model using voice commands to best visualize the 3D rendering overlaid on the patient’s anatomy.

**Results:**

We provide a video case demonstrating the use of the Hoth AR system for resection of a thoracic meningioma. The system was able to register the model to the patient accurately in under 30 s. It was used to accurately plan the incision preoperatively, determine levels for laminectomy intraoperatively, and for precise localization of the tumor prior to opening the dura.

**Conclusion:**

This case describes the clinical integration of a novel AR system for preoperative localization and intraoperative visualization during a resection of a thoracic meningioma. As AR systems continue to evolve and become more accessible, they are poised to become a standard tool in the surgical arsenal.

**Supplementary Information:**

The online version contains supplementary material available at 10.1007/s00701-025-06659-5.

## Introduction

Augmented reality (AR) is an emerging technology in neurosurgery that offers tremendous benefits in both academic training and clinical settings [7, 12]. By overlaying computer-generated images on the user’s view of the real world, AR provides three-dimensional (3D) reconstructions during complex surgeries. This capability allows for real-time and real-space engagement with or without complete operative exposure [15]. Over the past decade, interest in AR-assisted neurosurgery has grown rapidly.

AR has been explored for cerebral aneurysms, gliomas, and skull base meningiomas [5, 8, 9, 13]. It has been demonstrated that AR technology increases the precision of instrumentation [10, 11, 14]. It enables surgeons to visualize anatomical structures and pathological entities in three-dimensions, thereby offering a deeper understanding of the surgical landscape and facilitating surgical planning and execution [2]. Exploration of AR in spine surgery is relatively new, however. In spine surgery, methods such as intraoperative fluoroscopy or computed tomography (CT) based imaging guidance are used for localization, and they can be time-consuming, labor intensive, and can expose patients to significant amounts of radiation [5]. AR in spine surgery can potentially provide a solution to these problems.

In this technical note, we discuss the preoperative and intraoperative utilization of Hoth AR for spine surgery, and we discuss a representative case with operative video of a T8-T9 thoracic meningioma resection.

## Methods and materials

### Augmented reality system

**Fig. 1:**
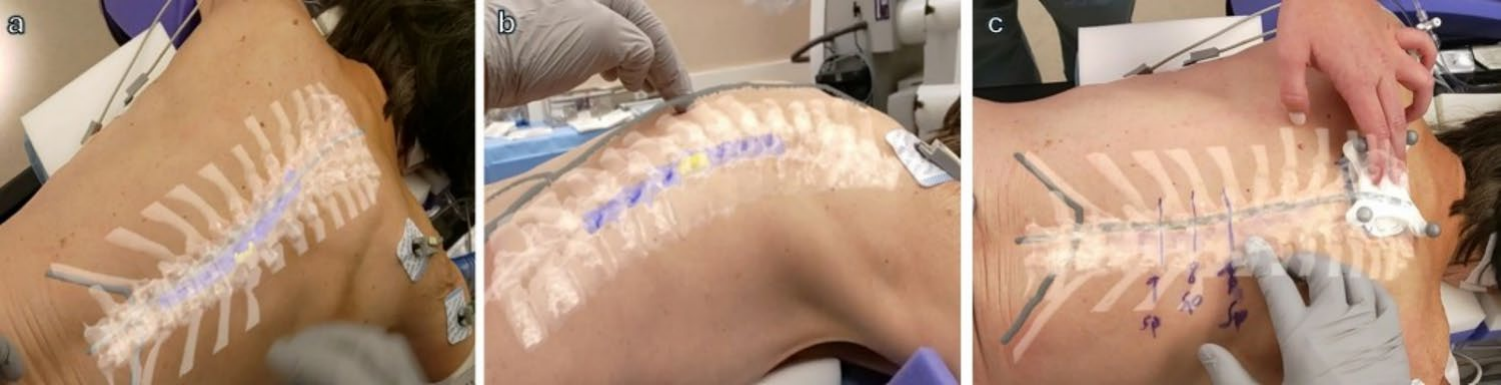
**a** and **b** Surgeon’s view through the AR headset of the 3D model registered onto the patient during registration and prior to incision with all model components visible. Model contains Bone (white), spinal cord (blue), tumor (yellow). **c** View of the model with bone only selected. Incision was planned by localizing the pedicles on the model

The Hoth Intelligence AR system (Hoth Intelligence, Philadelphia, PA, USA) is a mobile, head mounted display that utilizes a markerless registration process that is performed by tracking a tracing tool containing retroreflective spheres. For surgical planning, the system renders a 3D model of the surgical anatomy created from the patient’s imaging and stores the model in the headset. All AR visualization functionality is self-contained within the headset. To register the model to the patient for surgery, the headset tracks the tracing tool as the user traces along the reference points on the patient’s back. Additionally, the headset scans the surface of the patient’s back. By combining the surface scan with the tracing, the headset can align a 3D digital model with corresponding points on the preoperative scan resulting in the 3D anatomical model overlaid onto the patient’s back (Fig. 1). Currently, the AR system is being used as an investigational tool to assess its accuracy in localization in addition to current standard of care practices. Therefore, for spine surgery, fluoroscopic guidance with C-arm is performed after localization with the Hoth AR system at times when the provider would normally employ fluoroscopic localization.

### Ethical approval

Institutional Review Board approval was obtained for this work. The patient consented to the publication of their image.

### Operative use of augmented reality

A 3D AR model was rendered from preoperative magnetic resonance imaging (MRI), and the model consisted of the spine, labeled in white; the spinal cord, labeled in blue; and the tumor, labeled in yellow. Model registration is performed similarly to cranial surgery stereotactic navigation, where bony prominences are traced along the bridge of the nose and head with a tracking tool while an optical tracking device visualizes the tracing tool and a reference array simultaneously. However, unlike model registration for cranial surgery, there are less definable bony prominences in spine surgery, particularly in obese patients where the spine is difficult to palpate. Therefore, we predefined reference points to be used for patient registration and model overlay along the spinous processes and the 12th ribs. These reference points are seen as the gray line in the model in Fig. 1. Also, unlike standard stereotactic systems where the tracking cameras are in a separate console, the Hoth AR system tracking cameras are on the headset itself, so no separate console or camera system is required. Once the patient was asleep and positioned prone, with the headset on, we registered the model by tracing the reference points as described previously along the spinous processes and 12th rib. The cameras within the headset track the tracing tool tracker balls and simultaneously scan the back. The scanning of the back serves as the “reference array” for the model. A physical reference array with tracker balls can be used by taping the array to the patient’s back away from the incision if the scan of the native anatomy for reference is unsuccessful, however it was determined to not be needed in this case so it was removed prior to the start of the case. After completion of the registration process, the AR headset overlayed the model on the patient while viewing through the headset goggles (Fig. 1). By analyzing the bone elements of the model, the incision was designed by identifying the pedicles at the level of the tumor. The system does not yet have the capability to measure accuracy of the model quantitatively as it does not have a validated algorithm to calculate expected position of the model to actual positioning based on tracking the tracing tool. Therefore, standard localization with C-arm fluoroscopy was then performed to confirm the accuracy of the model and the planned incision.

**Fig. 2:**
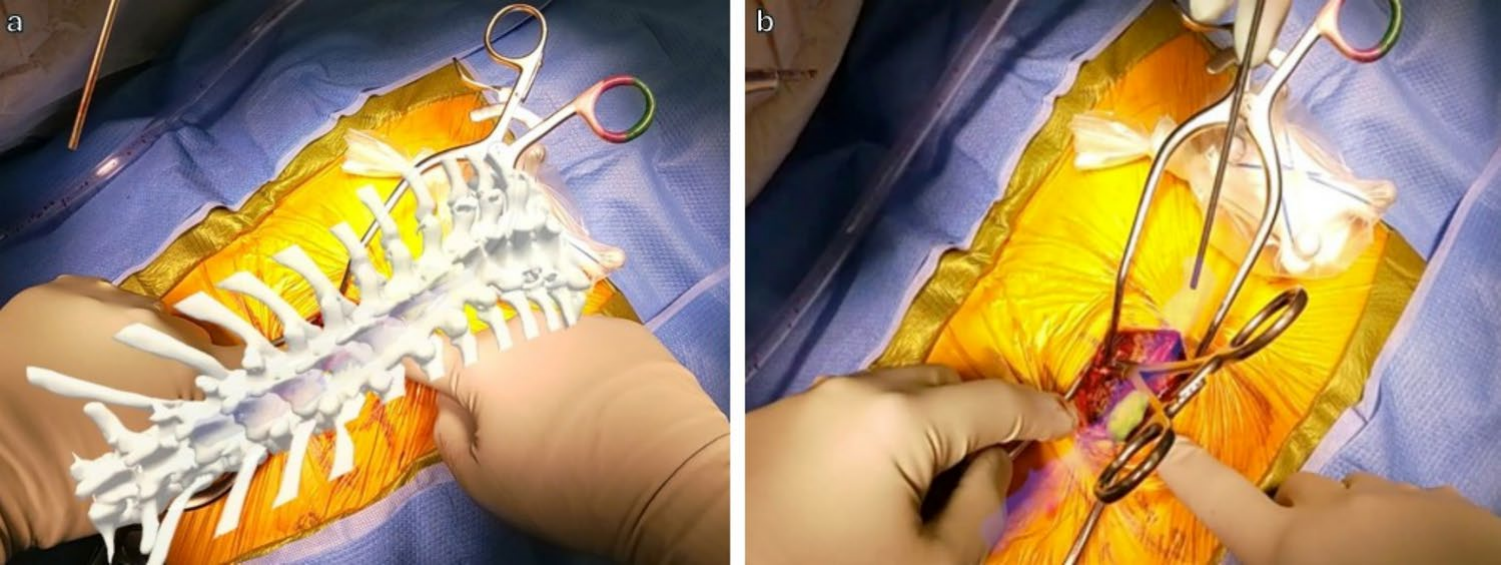
**a** Surgeon’s view through the AR headset of the 3D model after exposure of the spine viewing only the bony elements of the model for level localization. **b** View of the exposed spine after subtracting the bony elements from the model view. The spinal cord (blue) and tumor (yellow) are made transparent

While operating, we simultaneously wore the headset and surgical loupes. We look straight ahead to look through the headset goggles, and we turn our gaze downwards to look through loupe magnification. While exposing the spine, the 3D model was toggled to “off” with voice commands to allow us to focus with loupe magnification. After exposure, we used voice commands to toggle the 3D model “on” through the headset goggles (Fig. 2). We used voice commands to toggle the transparency and on–off of each component of the model to best visualize it over the exposed spine. The model was again used to determine the correct level of the spine and location of the tumor under the exposed bony elements. The spinal level was again confirmed using C-arm fluoroscopy.

**Fig. 3:**
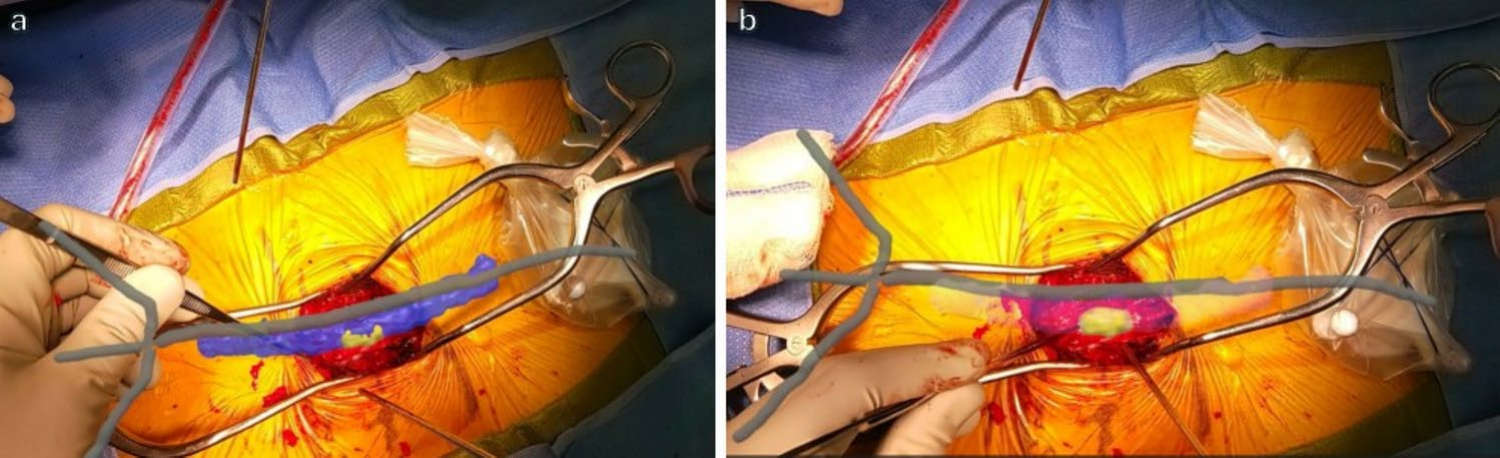
**a** Surgeon’s view through the AR headset of the 3D model after exposure of dura. Spine element of the model is subtracted here and only spinal cord (blue) and tumor (yellow) are seen. **b** Similar view but with the spinal cord and the tumor made transparent to plan the durotomy

Next, the model was toggled “off” again, and the laminectomies were performed through loupe magnification. Once the dura was completely exposed, the model was toggled “on” and viewed through the headset to visualize the tumor over the exposed dura (Fig. 3); the vertebrae were subtracted from the model with voice commands to optimize visualization. Aided by the headset, the durotomy was planned. At this point, the AR headset was removed, and the durotomy and tumor resection were performed via microscopy.

## Results

### Illustrative case

**Fig. 4:**
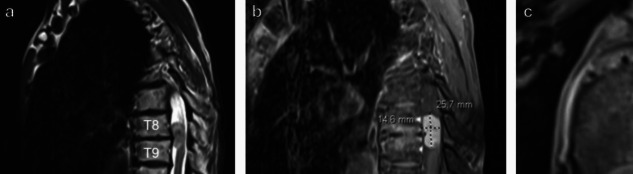
**a** Sagittal T2, **b** sagittal T1 post-contrast, and **c** axial T1 post-contrast MRI of the thoracic spine demonstrating the homogeneously enhancing lesion at T8 and T9 with severe spinal cord compression and displacement to the left

**Fig. 5:**
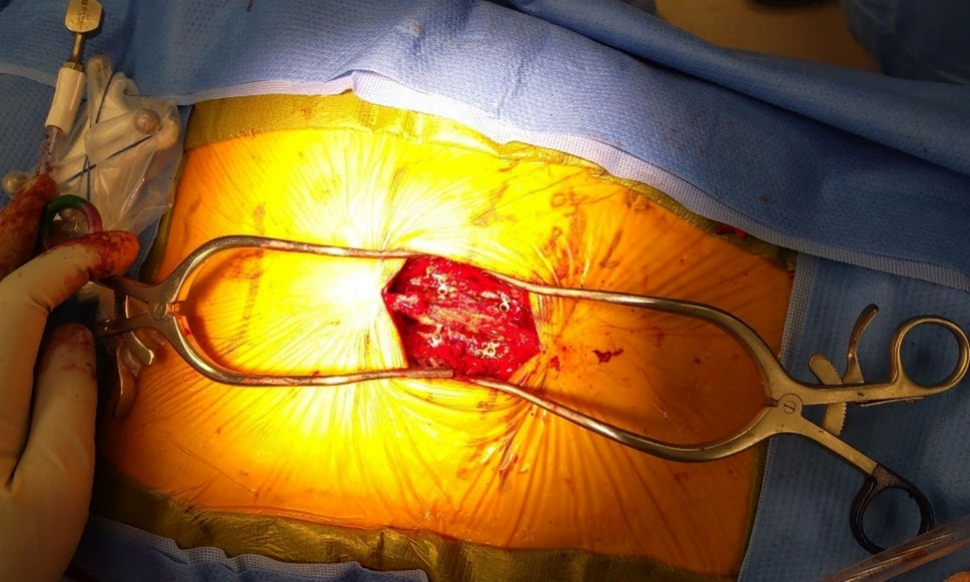
Operative view of completed T8-9 laminoplasty

A 69-year-old female presented with right lower extremity decreased sensation and shooting pain to the right lateral thigh. She denied lower extremity weakness or bowel/bladder changes. Physical exam demonstrated full strength in lower extremities and bilateral lower extremity hyperreflexia. MRI of the lumbar and thoracic spine showed L4-5 spondylolisthesis and lateral recess stenosis as the etiology of her radiculopathy and an intradural, extramedullary, well-circumscribed, homogeneously enhancing lesion at T8 and T9 with severe spinal cord compression concerning for meningioma (Fig. 4). She underwent a T8-9 laminoplasty (Fig. 5) for resection of the intradural extramedullary mass with AR assisted neuronavigation (Video 1).

### Outcome

The Hoth AR system was highly effective for preoperative planning and intraoperative navigation. As it is just a headset, the system does not take up any operating room space, and it was able to register the 3D model to the patient in under 30 s. The headset is lightweight and was able to comfortably be worn simultaneously with loupes without impairing visualization. The system was accurate in that it was able to correctly localize pedicles to plan the surgical incision, and it was able to correctly determine levels of the exposed spine, as these were both confirmed with fluoroscopy. No correction of misalignment of the rendered model was necessary. Furthermore, the system was effective in visualizing the tumor underneath the exposed dura in planning the durotomy.

**Fig. 6:**
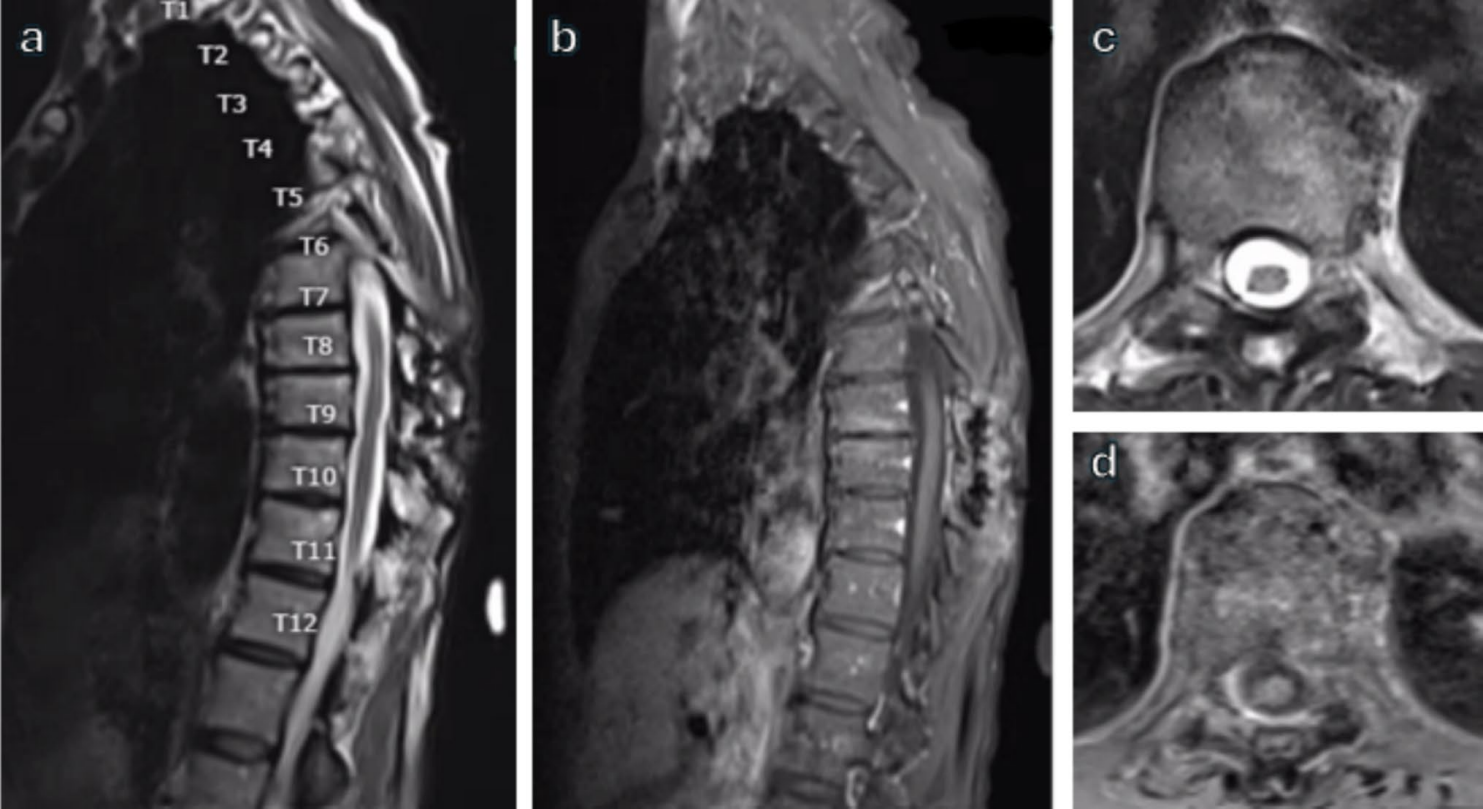
**a** Sagittal T2, **b** sagittal T1 post-contrast, **c** axial T2, and **d** axial T1 post-contrast MRI of the thoracic spine at 6 month postoperative visit demonstrating no residual or recurrent tumor

The postoperative course was uneventful. The patient recovered from surgery well and exhibited full strength in her lower extremities. She was discharged home on postoperative day 3. She was seen at 3- and 6-month follow-ups and had continued to recover very well and had returned to running. She had a repeat MRI of the thoracic spine with and without contrast by her 6 month follow up visit that demonstrated no residual or recurrent tumor (Fig. 6). Final pathology was consistent with meningioma World Health Organization Grade I.

## Discussion

We present our first spine surgery experience with the Hoth Intelligence AR system. Our results with the device emphasize the substantial advantages of AR-guided spine surgery operations, especially in terms of increasing precision and decreasing errors during the process. Prior studies have shown that AR enhances visualization in cases with complex anatomy, facilitating surgical precision and decision-making [3, 4]. Enhanced visualization afforded by AR proved to be valuable for neurosurgeons and researchers as well regarding complex instances with tumors in difficult-to-access areas [3, 6]. In our present experience, AR guided systems are less space occupying and quicker to use than a large C-arm machine for spinal localization with similar accuracy. As an emerging technology, AR will likely continue to improve to integrate even more seamlessly into the operating room workflow, with increasingly improving accuracy and intra-operative visualization of the 3D model as the technology continues to improve.

Furthermore, we believe that AR technology as a potential substitute for C-arm fluoroscopy can aid in minimizing radiation exposure, ensuring an environment that is safer for both patients and medical staff [1, 3, 6]. Conventional fluoroscopy also requires surgeons to wear lead aprons throughout the entire procedure, which can cause not only discomfort and increased fatigue, but also long-term health issues [16]. AR has the potential to significantly reduce or even eliminate such needs. The AR headset is easy to adapt into a surgeon’s practice as it is lightweight, comfortable, and it can easily be worn simultaneously with surgical loupes without compromising vision.

Despite the various benefits AR can bring to spine surgeries, there remain several limitations that are important to address. Integration of AR technology into the operating room requires substantial costs and technical training. Workflow adaptations and comprehensive education are needed for it to become fully integrated into daily practice. In spine surgery compared to cranial, the lack of prominent landmarks and reference points in the back poses a significant challenge to AR alignment and may necessitate manual adjustments. Though there was satisfactory overlay of augmented visuals over our patient’s anatomy, the Hoth headset can perform stepwise movement in any plane to perfect the alignment of the model, which does somewhat ameliorate this limitation. Lastly, the ability to project augmented visuals through surgical loupes and a microscope are not yet feasible, and as such, there is limited utility in using the headset for microdissection and resection, particularly in the case of intramedullary lesions.

## Limitations

We have provided our technical experience with a single device in a single institution with a single patient. More robust studies are required to assess AR in spine surgery scenarios including outcome and accuracy analyses and comparisons with other devices, surgeons, and pathologies.

## Conclusion

AR is a promising new technology which has the potential to provide benefits to both surgeons and patients. In our experience, the Hoth AR system integrates easily into the spine surgery workflow with a comfortable headset, rapid registration and intraoperative usability, and accuracy similar to C-arm fluoroscopy.

## Supplementary Information

Below is the link to the electronic supplementary material.

ESM1 Video 1: Case overview. This video demonstrates video footage of the surgeon’s view through the headset during preoperative and intraoperative use of the AR technology (MP4 276 MB)

## Data Availability

No datasets were generated or analysed during the current study.
